# Psychometric Properties of a Survey of Knowledge and Attitude Change in Residential Staff Receiving Training in Trauma-Informed Care: The Modularized Think Trauma Evaluation Questionnaires

**DOI:** 10.1007/s40653-025-00752-8

**Published:** 2025-09-10

**Authors:** Patricia M. Garibaldi, Neil Jordan, Cassandra Kisiel, Alysha D. Thompson, Tracy Fehrenbach

**Affiliations:** 1 Department of Psychiatry & Behavioral Sciences, Northwestern University Feinberg School of Medicine, Chicago, IL USA; 2 Departments of Preventive Medicine and Medical Social Sciences, Northwestern University Feinberg School of Medicine, Chicago, IL USA; 3 Department of Psychiatry and Behavioral Medicine, Department of Psychiatry and Behavioral Sciences, Seattle Children’s Hospital, University of Washington, Seattle, WA US

**Keywords:** Confirmatory factor analysis, Psychometrics, Children, Adolescents, Adverse childhood experiences

## Abstract

Trauma-informed care has received increased attention in the scientific literature and clinical practice (Becker-Blease, 2017; Purtle, 2020; Stokes et al., 2024); however, evaluation of the implementation and effectiveness of these efforts is limited (Purtle, 2020). This study addresses this gap by exploring the psychometric properties of the Modularized Think Trauma Evaluation Questionnaires (M-TTEQs). The M-TTEQs were developed to assess frontline residential staffs’ trauma-informed knowledge and attitudes before and after receiving Think Trauma, a National Child Traumatic Stress Network (NCTSN) training curriculum that consists of 4 discrete modules. This paper utilizes data from 1807 staff members at 20 Illinois child welfare residential care facilities who received Think Trauma training between 2020 and 2024. The internal consistency of M-TTEQs was assessed using Cronbach’s alpha, with results indicating strong internal consistency across all pre- and post-surveys (α values between 0.88 and 0.95), supporting the reliability of the measures. A subset of 155 participants who completed all 4 pre-training, and 153 participants who completed all 4 post-training M-TTEQs were included in confirmatory factor analyses (CFA) assessing the measures’ construct validity. CFA models demonstrated acceptable fit indices, indicating that the surveys measured the intended constructs for each module. Despite these acceptable psychometric properties, some items showed weaker factor loadings, particularly reverse-worded questions, suggesting the need for further refinement. This study contributes to the trauma-informed care literature by providing a tool with acceptable reliability and construct validity for assessing knowledge and attitude change related to the Think Trauma training curriculum.

## Introduction

Trauma-informed care at the agency or system level refers to approaches that (1) realize the widespread impact of trauma, (2) recognize the signs and symptoms of trauma, (3) respond by fully integrating knowledge about trauma into policies and organizational functioning, and (4) actively resist non-therapeutic re-traumatization (Substance Abuse and Mental Health Services Administration (SAMHSA), 2014). In contrast to trauma-focused clinical interventions (e.g., Trauma-Focused Cognitive Behavioral Therapy) that are delivered by mental health clinicians, trauma-informed approaches can be employed by a variety of staff and stakeholders (e.g., frontline and support staff, administrative leadership; Kisiel et al., 2024). Trauma-informed approaches are applicable to all people-facing settings and are particularly important in systems that serve individuals with elevated rates of trauma exposure and traumatic stress symptoms, such as the child welfare and juvenile justice systems. Research indicates that by the time of system entry, youth in the juvenile justice system have experienced an average of 5 types of traumatic events (e.g., traumatic loss, physical abuse, community violence) (Dierkhising et al., 2013) and youth in the child welfare system experience an average of 2 traumatic events, although many experience 3 or more (Garcia et al., 2017). In response to more widespread understanding of trauma’s impact on emotional and behavioral functioning (Kleber, 2019), there have been legislative and organizational efforts to embed trauma-informed approaches in settings that serve these youth. For example, the US Family First Prevention Services Act provides federal reimbursement to child welfare residential facilities practicing trauma-informed care (Text-H.R.1892-115th Congress, 2018). These legislative and policy efforts are indicated given the high rates of trauma exposure in these spaces (Garibaldi et al., 2024) and growing evidence supporting the effectiveness of trauma-informed approaches in the child welfare system (Bailey et al., 2019; Bartlett et al., 2018; Zhang et al., 2021).

The evidence associating trauma-informed care with improved wellbeing for youth involved in the child welfare system (Zhang et al., 2021) supports use of these practices; however, there are many gaps in our current understanding of the implementation, dissemination, and effectiveness of trauma-informed approaches. To further define and assess trauma-informed care, Hanson and Lang (2016) proposed 3 core domains of trauma-informed care in child welfare contexts through a synthesis of the peer-reviewed literature, resources created by nationally established organizations (e.g., the National Child Traumatic Stress Network), and input from over 400 providers. They found 3 key components for the implementation of trauma-informed care in the child welfare system: (1) workforce development (e.g., training, awareness, staff wellness), (2) trauma-focused services (use of evidence-based trauma interventions, screening, and assessment), and (3) the organizational environment and practices (e.g., safety in physical setting, collaboration, policies). Hanson and Lang (2016) also found that while these key components had agreement among multiple studies, providers, and national resources, about 50% of the over 400 providers surveyed indicated that their institutions did not formally evaluate their trauma-informed efforts. This highlights that while workforce development and staff training are critical to trauma-informed care, and many agencies are pursuing training in this area, there is a widespread lack of rigorous evaluation of trauma-informed approaches, which may result in the investment of resources in efforts that do not have the intended outcomes. These findings are echoed by Stokes and colleagues’, 2024 meta-analysis of trauma-informed care in pediatric residential and inpatient settings. This study notes barriers to widespread adoption and evaluation of existing evidence-based, trauma-informed models, including the lack of uniformity and detailed descriptions across models and approaches. Additionally, some models with strong evidence bases (e.g., the Sanctuary Model and the Attachment, Regulation & Competency framework) can be difficult to implement and evaluate due to significant financial costs and time commitments (Stokes et al., 2024).

Think Trauma is a trauma-informed training and implementation program that addresses shortcomings of other trauma-informed models, including lack of uniformity and evaluation processes, and high financial cost (Pickens et al., 2020). Think Trauma is a freely available, standardized trauma-informed care training curriculum that was developed through the National Child Traumatic Stress Network (NCTSN). The program is unique in that it is minimally burdensome, organized into modules allowing for flexible implementation, designed for use in residential settings, and written for frontline staff as the primary audience. There is some evidence for Think Trauma’s effectiveness, with one study showing that youth have significant decreases in trauma symptoms and incident reports after staff in residential juvenile justice settings received Think Trauma training and youth received trauma-focused group therapy (Trauma and Grief Component Therapy for Adolescents; Olafson et al., 2018). Olafson and colleagues’ (2018) study is encouraging as it showed widespread Think Trauma training for frontline staff was feasible. However, they did not parse apart the impact of Think Trauma versus the trauma-focused group intervention. This is aligned with many of the common challenges in the trauma-informed care literature, which include non-experimental study designs, relatively short follow-up periods, high rates of attrition, small sample sizes, and difficulty distinguishing the effects of organizational-level trauma-informed care initiatives in settings where several initiatives and interventions are being implemented concurrently (Bailey et al., 2019; Bryson et al., 2017; Bunting et al., 2019; Purtle, 2020).

This study will address some of these common methodological shortcomings and contribute to the limited body of literature about the evaluation of trauma-informed care. We examined the psychometric properties of questionnaires developed to measure the effectiveness of the Think Trauma curriculum in increasing trauma-informed knowledge and attitudes in residential staff serving youth in the Illinois child welfare system. The focus on trauma-informed knowledge and attitude change is important given the literature indicating that improvements in these areas are associated with individual and organizational willingness to adopt trauma-informed practices (Sundborg, 2019). Further, it is well established that behavior, and particularly behavioral change, is influenced by attitudes and perceived abilities to enact interventions’ target behaviors (Ajzen, 2011). Thus, this study seeks to explore: (1) the usability of the Modularized Think Trauma Evaluation Questionnaires (M-TTEQs), measures that evaluate trauma-informed knowledge and attitudes before and after Think Trauma training, (2) metrics of reliability and validity of this measure, and (3) recommendations for future evaluation of Think Trauma and other trauma-informed care trainings.

## Methods

### Participants

Participants included 1807 staff at 20 Illinois child welfare residential care facilities that received Think Trauma training between December 2020 and September 2024. These 1807 staff were individuals who completed at least one evaluation survey when they were trained in Think Trauma. Responses from 155 staff who completed all 4 pre-training and 153 staff who completed all 4 post-training evaluation surveys were used in the confirmatory factor analyses to establish the surveys’ construct validity.

### Procedures

**Fig. 1:**
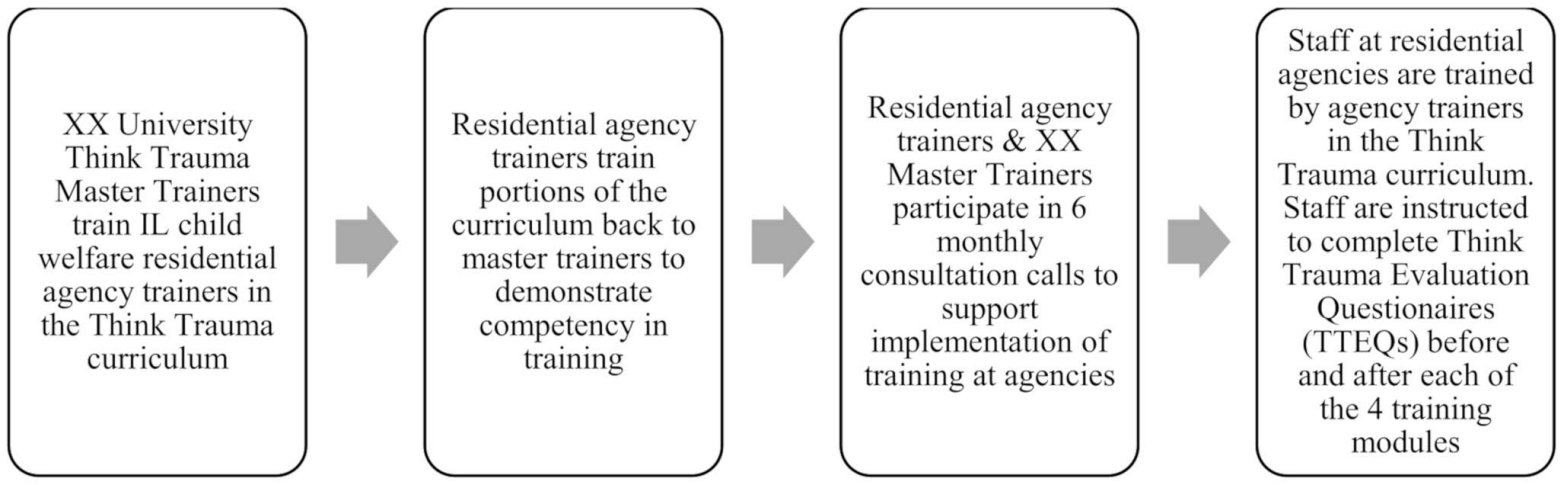
Training of Trainers (TOT) process

The participants in this study were trained in Think Trauma by individuals from their residential agency who completed a Think Trauma Training of Trainers (TOT) program. Master trainers affiliated with Northwestern University regularly deliver Think Trauma TOT to staff who are interested in becoming trainers at their agency. Annually across the 4 years of this study, each TOT included an average of 8 residential agencies, with 3–5 representatives per agency. The training was provided at no cost to all participating residential agencies. The TOT lasted approximately 4 days totaling 18 hours of training, “teach back” sessions during which agency trainers administered portions of the curriculum to the master trainers to demonstrate competency and receive feedback, and 6–8 months of consultation calls with master trainers to provide training and implementation support (see Fig. 1).

The participants in this study received Think Trauma training from their respective agency trainers and completed trauma-informed knowledge and attitude surveys (Modularized Think Trauma Evaluation Questionnaires or M-TTEQs) before and after each training module. Think Trauma training consists of 16–18 h of curriculum across 4 discrete modules: (1) Trauma Exposed Youth and System Involvement, (2) Trauma’s Impact on Development, (3) Trauma in Context and Coping, and (4) Trauma and Staff Wellness. The Think Trauma 2nd Edition, which was used in this study, was updated prior to its release in 2020 to reflect scientific advances and research accumulated since the creation of the first edition in 2010. The updates included a more enhanced focus on complex trauma throughout the modules and highlighted the role of historical, intergenerational, racial, and system-induced traumas for many youth in residential care. Further, the 2nd edition places a greater emphasis on implementation, cross-setting application, and staff wellness, offering a deeper dive into the context of workplace stress and opportunities to fully integrate trauma-informed and wellness practices for staff into the process of creating a trauma-informed agency. While the Think Trauma training curriculum was originally designed for use in juvenile justice residential settings, the curriculum content is applicable to both child welfare and juvenile justice residential settings and populations. Master trainers affiliated with Northwestern University made minor adjustments to the curriculum to adapt the content of the training to the child welfare system (e.g., ensuring statistics about trauma exposure captured child welfare involved youth, updating case examples). Agency trainers were taught how to administer the M-TTEQ evaluations, and the Northwestern University team intermittently reminded trainers of evaluation procedures throughout the consultation period. Additionally, links and QR codes providing access to the M-TTEQs are embedded directly into the curriculum at the beginning and end of each module.

While the intention is for participants to receive all 4 modules of the Think Trauma training, it is common for participants to be trained in some, but not all the modules, and for trainers to train either a single or partial module at a time given the length of the curriculum and related system constraints (e.g., units needing 24/7 staffing, staffing shortages) present within child welfare residential settings. For this reason, using the module-based version of the TTEQ tool for Think Trauma curriculum was critical. This project received a “Not Human Research” designation from the Northwestern University IRB.

### Measures

The Modularized Think Trauma Evaluation Questionnaires (M-TTEQs) are an adaptation of the original TTEQ tool, a 45-item evaluation measure developed for the original Think Trauma Curriculum (Marr et al., 2015). Each M-TTEQ has 10, 5-point (response options: strongly agree, agree, neutral, disagree, or strongly disagree) Likert scale questions and is administered before and after Think Trauma training to assess knowledge and attitudes related to trauma-informed care. The questions in each M-TTEQ align with the content of the corresponding module of the 2nd Edition of the Think Trauma Curriculum (Pickens et al., 2020). Cover pages added to the M-TTEQs captured respondents’ professional role, duration in their professional role, perceived degree of existing trauma knowledge, and if they had previously received Think Trauma training. Surveys were administered using Qualtrics software. See Appendix A for the survey items included in each questionnaire. The M-TTEQs were developed in response to feedback from trainers and participants on the original 45-item TTEQ (Marr et al., 2015), which was administered on paper and pencil once at the beginning of module 1 and the end of module 4. The original TTEQ was challenging to use because (1) many agencies trained one module at a time, sometimes with extended time between trainings due to staffing and coverage issues, and (2) some agencies did not train in sequential order, with a subset of agencies choosing to train module 4 about staff wellness first. In response to this feedback, 5 core developers and expert trainers in the Think Trauma original and 2nd editions collaborated to create the M-TTEQs. These developers were clinical psychologists with expertise in child trauma and trauma-informed care. They independently compared the original TTEQ with the 2nd edition of the Think Trauma curriculum, identifying questions that corresponded to the most recent edition. The developers then collaboratively decided which items to keep from the original TTEQ, striving to keep the overall number of items low to decrease respondent burden. Although some original items were modified for inclusion in the M-TTEQ, others were eliminated. Items were maintained in their original or edited version if at least 4 of the 5 curriculum developers voted in favor of the survey item. This group of core developers also developed 8 new questions – 2 for module 1, 1 for module 2, 4 for module 3 and 1 for module 4 – to better capture the updated content and focus on implementation present in the 2nd edition of the Think Trauma curriculum.

### Data Analysis

All analyses examining the psychometric properties of the completed M-TTEQs were conducted using R (R Core Team, 2024). Data were extracted from Qualtrics, and we deleted responses that were (1) incomplete (e.g., completed demographic items but did not complete any content questions), or (2) from individuals who completed the same survey more than once within 6 months (e.g., a respondent completing a pre-training Module 1 TTEQ twice in 6 months). Between 7% and 12% of survey responses for each module were deleted in the data cleaning process. To measure internal consistency, Cronbach’s alphas for each M-TTEQ were calculated using all remaining data. To measure construct validity, we completed confirmatory factor analysis (CFA) (Brown, 2006a) for all (1) pre-training surveys, and (2) post-training surveys to assess whether the items included in each module’s survey were measuring the same intended construct. Due to the survey response items falling along a Likert scale with data skewed toward “agree” responses, the data were treated as ordinal. We completed confirmatory factor analyses (CFA) using (1) M-TTEQ data from 155 participants who completed all 4 pre-training surveys and (2) M-TTEQ data from 153 participants who completed all post-training surveys. The CFA models had 4 factors, with a factor for each module, allowing for correlations between the factors. There was 1 CFA conducted for all pre-training M-TTEQs, and 1 CFA conducted for all post-training M-TTEQs. Due to the ordinal nature of the data, we used a weighted least square estimator (WLSMV) for the CFAs based on polychoric correlation matrices (Brown, 2006b). The fit statistics used to determine the appropriateness of the models were (1) the root mean square error of approximation (RMSEA; recommended to be ≤ 0.08 for an acceptable fit), (2) the Comparative Fit Index (CFI; recommended to be ≥ 0.95), and (3) the Tucker-Lewis Index (TLI; recommended to be ≥ 0.95; Brown, 2006a).

## Results

The number of responses for each module’s pre and post M-TTEQ ranged from 446 responses to 802 responses (Table 1) and were collected from 1807 unique individuals. 21% of respondents were direct service/milieu workers, 14% were mental health clinicians, 12% were caseworkers, 11% were youth workers/recreation workers, 11% had a role that fell into “other,” and 9% were supervisors (Table 2). 33% of respondents had been in their role/profession for less than a year, 23% for 1–3 years, 9% for 4–6 years, 9% for 7–10 years, 6% for 11–15 years, and 12% for more than 15 years. Most participants self-reported “much” to “a great deal” of knowledge about trauma at baseline with 13% reporting “not much” or “a little” trauma knowledge, 28% reporting “some”, 29% reporting “much”, and 24% reporting “a great deal.” Most (57%) reported not having received any previous training in Think Trauma, 28% reported past Think Trauma training, and 9% reported being unsure.

**Table 1:** Number of surveys submitted

	Pre-Training Surveys	Post-Training Surveys
Module 1: Trauma & System Involvement	802	708
Module 2: Trauma’s Impact on Development	653	518
Module 3: Trauma in Context & Coping	446	485
Module 4: Trauma & Staff Wellness	678	578

**Table 2:** Respondent characteristics (*N* = 1807)

Job Title^1^	
Direct service/milieu worker	380 (21%)
Mental Health Clinician/supervisor	252 (14%)
Caseworker/casework supervisor	213 (12%)
youth worker/recreation	195 (11%)
Other (unspecified)	191 (11%)
Supervisor	159 (9%)
Administrator/Director	82 (4%)
Teacher	66 (4%)
Administrative Support	53 (3%)
Unit Manager	49 (3%)
Foster Parent	24 (1%)
Maintenance & Facilities Support	21 (1%)
Medical/Nursing	21 (1%)
Missing	101 (6%)
**Time in Profession** ^1^	
Less than 1 year	594 (33%)
1–3 years	415 (23%)
4–6 years	212 (9%)
7–10 years	154 (9%)
11–15 years	113 (6%)
More than 15 years	217 (12%)
**Self-Reported Baseline Trauma Knowledge** ^1^	
Not much	71 (4%)
A little	156 (9%)
Some	503 (28%)
Much	519 (29%)
A great deal	438 (24%)
**Prior Think Trauma Training** ^1^	
Yes, at current agency	370 (20%)
Yes, at a previous agency	146 (8%)
No	1035 (57%)
Unsure	154 (9%)

Note: ^1^ n (%)

There were 155 of the respondents (8.6% of total respondents) who completed all (4) pre training M-TTEQs and 153 (8.5% of total respondents) who completed all (4) and post M-TTEQs. Data from these respondents were used to assess construct validity via CFA. Only 97 (5.4% of total respondents) completed all (8) pre and post M-TTEQ surveys. Based on Chi-Square statistical tests, there were no statistically significant differences in job titles, duration of experience, prior experience with Think Trauma, or self-reported baseline trauma knowledge between the individuals who completed all 8 M-TTEQs and those who completed 1–7 M-TTEQs.

**Fig. 2:**
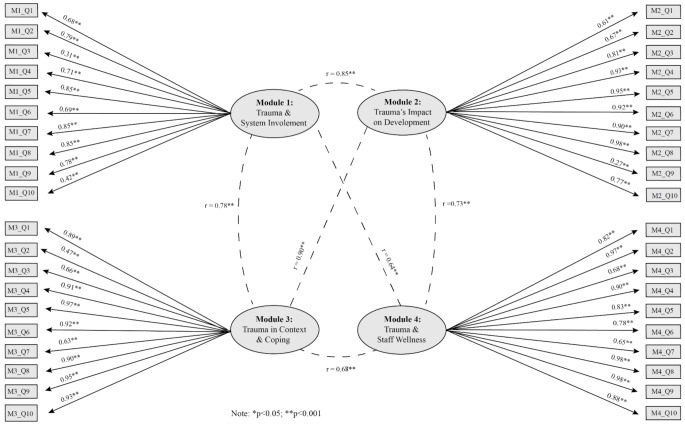
Standardized factor loadings and inter-factor correlations for the pre-training M-TTEQ confirmatory factor analysis

**Fig. 3:**
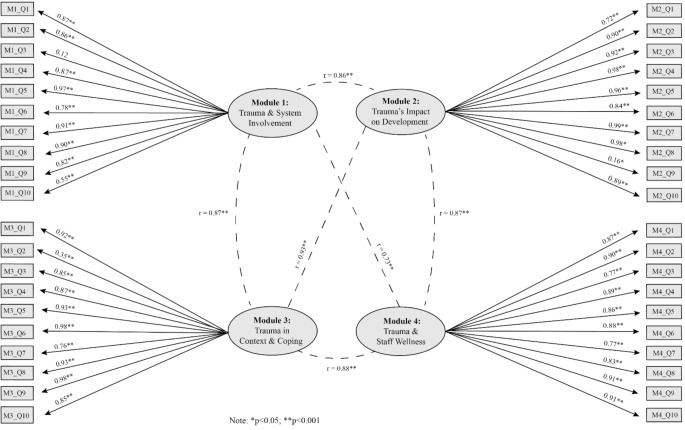
Standardized factor loadings and inter-factor correlations for the post training M-TTEQ confirmatory factor analysis

To assess the reliability, via internal consistency, of the Modularized TTEQs, we calculated Cronbach’s alpha for each pre and post M-TTEQ. Results found good to excellent internal consistency across all surveys, with α values ranging from 0.88 to 0.95 (Taber, 2018; Table 3). Four-factor models were tested in both CFAs to explore whether the questions corresponding with each module were measuring the same underlying constructs. The models were found to be acceptable for the pre training M-TTEQs and post training M-TTEQs (Table 4). For all 8 factors (4 factors for the pre M-TTEQs and 4 for the post M-TTEQs), all but one of the corresponding items demonstrated statistically significant factor loadings, and most items had standardized factor loadings greater than 0.50 (Hair, 2010). Question 3 for module 1 post-training M-TTEQ was the only item that had a non-significant factor loading. For the pre (Fig. 2) and post training (Fig. 3) surveys, questions 3 and 10 in module 1, question 9 in module 2, and question 2 in module 3 had factor loadings lower than the acceptable criteria of 0.50 (Hair, 2010). The factors also demonstrated strong, positive correlations with each other, with correlations ranging from 0.64 to 0.90 between factors for the pre-training surveys, and 0.73–0.93 between factors for the post-training surveys. This suggests a strong degree of overlap in the constructs between the factors, which is to be expected given that all modules focus on trauma-informed practices.

**Table 3:** Internal consistency of M-TTEQs

Survey	Cronbach’s α
Module 1 Pre-Training TTEQ	0.88
Module 1 Post-Training TTEQ	0.89
Module 2 Pre-Training TTEQ	0.93
Module 2 Post-Training TTEQ	0.95
Module 3 Pre-Training TTEQ	0.94
Module 3 Post-Training TTEQ	0.95
Module 4 Pre-Training TTEQ	0.95
Module 4 Post-Training TTEQ	0.92

**Table 4:** Goodness-of-Fit statistics

Model	χ^2^	df	CFI^1^	TLI ^2^	RMSEA^3^	90% CI
CFA 4 factor, Pre M-TTEQs	1330	734	0.976	0.974	0.073	[0.066, 0.079]
CFA 4 factor, Post M-TTEQs	1265	734	0.986	0.985	0.069	[0.063, 0.075]

^1^CFI=Comparison Fit Index; ^2^TLI=Tucker-Lewis Index; ^3^RMSEA=Root Mean Square Error of Approximation

## Discussion

This paper illustrates that the M-TTEQs have sufficient reliability, as shown through measures of internal consistency, and have some evidence of construct validity, as demonstrated via CFA. The participants in this sample were primarily direct care staff, mental health clinicians, and caseworkers, with most having less than a year of experience. These trainee characteristics are expected as it is common for agencies to conduct trauma-informed trainings for the staff that have the most client-facing contact during employee orientation and onboarding. Of note, while most participants had not previously received Think Trauma training, most reported having at least some to a great deal of trauma knowledge at baseline, which may influence the degree to which their trauma knowledge and attitudes may change over time independent of the Think Trauma training. This relatively high level of perceived trauma knowledge at baseline can be understood in the context of the recent proliferation of research and general knowledge about trauma-informed care (Becker-Blease, 2017); this may reflect staff being highly knowledgeable, or overestimating their existing knowledge, about trauma prior to the training.

The data collection methods utilized in this training were successful, as demonstrated by the large amount of data collected, providing guidance to future trauma-informed care evaluation efforts. More specifically, this study suggests the benefit of using relatively brief, online surveys that can be completed on any internet-compatible device with corresponding QR codes and survey links directly embedded into the training curriculum. This survey methodology yielded significant differences in the number of surveys submitted for each module (Table 1), with there being more pre training than post training M-TTEQs submitted for 3 of the 4 modules. This could be due to respondent fatigue or trainers running out of time to facilitate the survey completion at the end of training sessions. The issue of survey fatigue, or non-response rates to surveys increasing due to greater survey demand or exposure, has been found across many settings and populations (De Koning et al., 2021; Porter et al., 2004). The differences in survey responses between modules also provides insight into this issue. The greatest number of surveys were submitted for modules 1 and 4, which is not surprising given that many agencies opt to start with one of these two modules: 1 (due to the foundational knowledge it presents) or 4 (to emphasize the importance of staff wellness). There were notably fewer surveys submitted for module 3, even at pre-, which could reflect agencies opting to skip or shorten that module. If future research ascertains that the information presented in module 3 about trauma in context or coping feels less relevant or compelling to residential staff, this would be important and may result in curriculum adjustments.

This study also provides suggestions for how to improve the M-TTEQs in the future. A few questions in the modularized TTEQ had lower levels of internal consistency and factor loadings, and their inclusion warrants reconsideration. Questions with weaker metrics include 1) module 1, item 3 (“Youth who I work with use their trauma as an excuse for their behavior”), 2) module 1, item 10 (“Trauma is the same as stress’); 3) module 2, question 9 (“mental health counseling is the only way to help youth become more resilient”), and 4) module 3, question 2 (“a youth always knows when he/she is being triggered”). While most of these questions were significantly associated with its module’s factor, they showed relatively low factor loading (< 0.50) and internal consistency. These questions represent 4 of the 5 questions across all surveys that were reverse worded, with the trauma-informed response aligning with the “disagree” end of the Likert scale. Reverse worded items have customarily been included in surveys to control for acquiescence bias, or the tendency for respondents to agree with survey items (Schriesheim & Hill, 1981). However, particularly when used in Likert scale surveys, there is growing evidence discouraging the inclusion of reverse worded questions. Reverse worded questions can be difficult for respondents to interpret and lead to heightened rates of mis-response that are unrelated to inattention or acquiescence bias (Swain et al., 2008). Further, reverse worded items can weaken the factor structure of surveys and reduce reliability (Zhang et al., 2016). Thus, it is not surprising that the reverse worded questions in the M-TTEQs had lower factor loadings and suggests that these questions should be modified to be positively worded (e.g., higher agreement aligning with trauma-informed principles).

While this study makes several contributions, it has limitations that necessitate consideration. First, this study has methodological weaknesses that limit the findings’ generalizability. This study did not include existing validated measures of trauma-informed knowledge and attitudes, which prevents us from fully establishing the M-TTEQ’s psychometric properties through exploration of additional types of validity (e.g., discriminant and concurrent validity). Additional limitations are presented by the small proportion of trained staff (5.4%) who completed all 8 M-TTEQs, as compared to the 1807 staff who completed at least one survey. While there were no statistically significant differences between these groups based on the data available (job title, years in their roles, self-reported trauma knowledge at baseline), the individuals who completed all 8 surveys may differ in unmeasured ways from the staff who completed fewer surveys. Future iterations of the surveys may include other demographic variables, such as respondent age, gender, or geographic location, to explore other potential differences in survey completion rates and M-TTEQ responses. Further, we are unable to report a true response rate because agency trainers report the number of people they trained separate from their submission of the M-TTEQs, and submission of the attendance data for the completed trainings was inconsistent.

Training agency staff using a TOT model has been found to be effective for dissemination of knowledge and information to health and social service providers (Pearce et al., 2012). The TOT method is particularly practical in the context of a large child welfare system as it allows for more rapid dissemination of the training across a large geographic area and facilitates capacity-building for local agencies and sustainability of the training in high-turnover environments. However, the use of TOT models creates evaluation and research challenges as there is diminished ability to control many variables, including quality of and fidelity to the training and evaluation processes relative to traditional training models. Another important limitation is that the surveys asked only about self-reported knowledge and attitudes immediately before and after the trainings. Self-perceived trauma knowledge can be impacted by a person’s desire to be perceived positively. A final limitation is presented by this survey being tailored to the Think Trauma curriculum content and structure, with the applicability of the surveys to trauma-informed trainings in general being unclear.

Despite these limitations, this study contributes to the literature by establishing the basic psychometric properties of a tool that can be used by the wide array of individuals and agencies implementing the Think Trauma curriculum both nationally and internationally. More specifically, this study offers findings on internal consistency and aspects of construct validity for the Modularized TTEQs. Though additional research into the psychometric properties of the M-TTEQs (e.g., across different settings) will be beneficial, this study suggests that these brief and easy to administer surveys can effectively evaluate the impact of the Think Trauma curriculum on changes in staff knowledge and attitude among frontline child welfare residential care staff. This is a significant contribution given the lack of standardized evaluation methods, use of validated measures, and clearly defined procedures that is evident in much of the trauma-informed care literature. This study provides a foundation for future work examining if the Think Trauma training curriculum is associated with significant trauma knowledge and attitude changes for residential staff.

## Conclusions

This study explored the psychometric properties of a knowledge and attitude survey administered before and after a trauma-informed care training, Think Trauma, in child welfare residential care facilities in Illinois. These surveys, the Modularized Think Trauma Evaluation Questionnaires (M-TTEQs), were found to be feasible to administer as demonstrated by the large quantity of data submitted. The M-TTEQs include 10 pre- and post-training questions tailored to the content of the 4 training modules: (1) Trauma & system involvement, (2) Trauma’s impact on development, (3) Trauma in context and coping, and (4) Trauma and staff wellness. All the M-TTEQs demonstrated strong internal consistency with evidence of acceptable construct validity. This establishes initial evidence for validity and reliability of the M-TTEQs and provides the basis for research that examines the validity and reliability of the M-TTEQs in other settings. Together, this study established internal consistency and aspects of construct validity for the Modularized Think Trauma Evaluation Questionnaires and contributes to the growing evidence base about how to evaluate trauma-informed staff trainings.

## Data Availability

Research data are not shared.

## Appendix A: Modularized Think Trauma Evaluation Questionnaires (M-TTEQ) Survey Items

### Options for all Surveys

Strongly Agree, Agree, Neutral, Disagree, Strongly Disagree.

### Note

Reverse worded questions are bolded.

### Module 1 TTEQ Questions

1. Trauma can result in feeling “on guard” all of the time.
2. Sounds, smells, physical sensations, people, thoughts and even emotions can be trauma reminders for youth.
3. Youth who I work with use their trauma as an excuse for their behavior.
4. Helping youth understand and address their trauma will help them to act out less often and cope effectively.
5. Sometimes traumatic stress reactions can look like zoning out or not wanting to engage with others.
6. An event is traumatic only if the youth was physically injured in the event.
7. Youth can experience trauma reminders while receiving services in detention or residential treatment settings.
8. Everyone can have an important role in building resilience by connecting with youth, encouraging them to share their feelings, and helping them to identify healthy ways to cope.
9. Trauma can result in always being on the lookout for danger cues, such as thinking other people are angry, even when they are not.
10. Trauma is the same as stress.

### Module 2 TTEQ Questions

1. Trauma can result in youth being too trusting and unable to trust others.
2. Understanding trauma can improve a youth’s physical and emotional safety in residential settings.
3. Trauma affects youth brain development.
4. Trauma can result in difficulties managing anger and defensive attitudes towards others.
5. Understanding trauma can help me relate to and interact with youth in more effective ways.
6. Lack of consistently available caregivers during childhood can negatively impact youth development.
7. Trauma can lead to difficulties expressing emotions. Although youth may express anger, they actually may feel sad or afraid.
8. Trauma exposure can impact a youth’s relationship with friends or family.
9. Mental health counseling is the only way to help youth become more resilient.
10. Focusing on youth’s natural talents and strengths can facilitate healthy coping and empower youth.

### Module 3 TTEQ Questions

1. An increase in heart rate, blood pressure, and breathing are all examples of the body’s alarm response to trauma reminders.
2. A youth always knows when he/she is being triggered.
3. Youth who are homeless, have special needs, and identify as LGBTQ are at higher risk for trauma experiences.
4. Trauma-informed safety plans can be a useful collaborative tool between staff and youth to increase self-awareness and healthy coping.
5. A youth’s trauma experiences can strongly impact the way they think about themselves, others, and the world.
6. When systems responsible for protecting youth do not fully understand or recognize the impact of trauma, they can cause further harm.
7. Youth can be negatively impacted by traumatic events that occurred before they were born (e.g., slavery, the Holocaust).
8. Trauma reminders and calming behaviors should be included in a youth’s safety plan.
9. While often unrecognized, discrimination, poverty, and community violence are systemic adverse childhood experiences that can negatively impact youth functioning.
10. Trauma can result in maladaptive coping strategies for youth.

### Module 4 TTEQ Questions

1. Promoting staff wellness is the responsibility of the organization, supervisor, and each individual staff member.
2. Understanding trauma can help staff work together more effectively.
3. Stress at work can cause staff to discipline youth more severely.
4. Understanding the impact of trauma can improve job satisfaction.
5. Staff stress levels can impact the youth in their care.
6. Hearing about the trauma that youth have experienced can be traumatic for me and my coworkers.
7. Working with youth who have experienced trauma could have a negative impact on how staff see the world.
8. Physically restraining youth can be traumatic for staff.
9. Supervisors should help staff manage workplace stress.
10. Understanding trauma can help staff reduce the use of restraints in residential settings.

## References

[CR1] Ajzen, I. (2011). Behavioral interventions: Design and evaluation guided by the theory of planned behavior. In M. M. Mark, S. I. Donaldson, & B. Campbell (Eds.), *Social psychology and evaluation* (pp. 74–103). Guilford Press.

[CR2] Bailey, C., Klas, A., Cox, R., Bergmeier, H., Avery, J., & Skouteris, H. (2019). Systematic review of organisation-wide, trauma‐informed care models in out‐of‐home care (Oo HC) settings. *Health & Social Care in the Community*, *27*(3), e10–e22. 30033666 10.1111/hsc.12621

[CR3] Bartlett, J. D., Griffin, J. L., Spinazzola, J., Fraser, J. G., Noroña, C. R., Bodian, R., & Barto, B. (2018). The impact of a statewide trauma-informed care initiative in child welfare on the well-being of children and youth with complex trauma. *Children and Youth Services Review*, *84*, 110–117.

[CR4] Becker-Blease, K. A. (2017). As the world becomes trauma informed, work to do. *Journal of Trauma Dissociation*, *18*, 131–138. 10.1080/15299732.2017.1253401 28145820 10.1080/15299732.2017.1253401

[CR6] Brown, T. A. (2006a). Introduction to confirmatory factor analysis. *Confirmatory Factor Analysis for Applied Research, 40–102*, Guilford Press.

[CR5] Brown, T. A. (2006b). Data issues in CFA: Missing, non-normal, and categorical data. *Confirmatory Factor Analysis for Applied Research*, *363*, 411.

[CR7] Bryson, S. A., Gauvin, E., Jamieson, A., Rathgeber, M., Faulkner-Gibson, L., Bell, S., & Burke, S. (2017). What are effective strategies for implementing trauma-informed care in youth inpatient psychiatric and residential treatment settings? A realist systematic review. *International Journal of Mental Health Systems*, *11*, 1–16. 28503194 10.1186/s13033-017-0137-3 PMC5425975

[CR8] Bunting, L., Montgomery, L., Mooney, S., MacDonald, M., Coulter, S., Hayes, D., & Davidson, G. (2019). Trauma informed child welfare systems—A rapid evidence review. *International Journal of Environmental Research and Public Health*, *16*(13), 2365. 31277339 10.3390/ijerph16132365 PMC6651663

[CR9] De Koning, R., Egiz, A., Kotecha, J., Ciuculete, A. C., Ooi, S. Z. Y., Bankole, N. D. A., & Kanmounye, U. S. (2021). Survey fatigue during the COVID-19 pandemic: An analysis of neurosurgery survey response rates. *Frontiers in Surgery*, *8*, 690680. 34458314 10.3389/fsurg.2021.690680 PMC8388838

[CR10] Dierkhising, C. B., Ko, S. J., Woods-Jaeger, B., Briggs, E. C., Lee, R., & Pynoos, R. S. (2013). Trauma histories among justice-involved youth: Findings from the National child traumatic stress network. *European Journal of Psychotraumatology*, *4*(1), 20274. 10.3402/ejpt.v4i0.20274 PMC3714673 23869252

[CR11] Garcia, A. R., Gupta, M., Greeson, J. K., Thompson, A., & DeNard, C. (2017). Adverse childhood experiences among youth reported to child welfare: Results from the National survey of child & adolescent wellbeing. *Child Abuse & Neglect*, *70*, 292–302. 28668759 10.1016/j.chiabu.2017.06.019

[CR12] Garibaldi, P., Kisiel, C., Castillo, A., Mitchell-Adams, H., & Jordan, N. (2024). Direct care staff vacancies and adverse youth events in Illinois child welfare residential treatment during the COVID-19 pandemic. *Residential Treatment for Children & Youth*, 1–21.

[CR13] Hair, J. F. (2010). *Multivariate data analysis* (7th ed.). Prentice Hall.

[CR14] Hanson, R. F., & Lang, J. (2016). A critical look at trauma-informed care among agencies and systems serving maltreated youth and their families. *Child Maltreatment*, *21*(2), 95–100. 26951344 10.1177/1077559516635274

[CR16] Kisiel, C., Guarnaccia, U., Pinkerton, L., Garibaldi, P., & Agosti, J. (2024). Empowering transition age youth through trauma-informed, strengths-based, youth-centered, and anti-racist practices: Implementation of a virtual breakthrough series collaborative. *Children and Youth Services Review*, *163*, 107682.

[CR17] Kleber, R. J. (2019). Trauma and public mental health: A focused review. *Frontiers in Psychiatry*, *10*, 451. 31293461 10.3389/fpsyt.2019.00451 PMC6603306

[CR18] Marr, M., Surko, M., Storfer-Isser, A., Havens, J. F., Richardson, L., & Horwitz, S. M. (2015). Think trauma evaluation questionnaire: Factor structure and feasibility of large scale administration. *Journal of Child & Adolescent Trauma*, *8*, 229–235.

[CR19] Olafson, E., Boat, B. W., Putnam, K. T., Thieken, L., Marrow, M. T., & Putnam, F. W. (2018). Implementing trauma and grief component therapy for adolescents and think trauma for traumatized youth in secure juvenile justice settings. *Journal of Interpersonal Violence*, *33*(16), 2537–2557. 26872505 10.1177/0886260516628287

[CR20] Pearce, J., Mann, M. K., Jones, C., Van Buschbach, S., Olff, M., & Bisson, J. I. (2012). The most effective way of delivering A Train-the‐Trainers program: A systematic review. *Journal of Continuing Education in the Health Professions*, *32*(3), 215–226. 23173243 10.1002/chp.21148

[CR21] Pickens, I., Marrow, M., & Benamati, J. (2020). *Think trauma: A training for working with justice- involved youth*. National Center for Child Traumatic Stress.

[CR22] Porter, S. R., Whitcomb, M. E., & Weitzer, W. H. (2004). Multiple surveys of students and survey fatigue. *New Directions for Institutional Research*, *2004*(121), 63–73.

[CR23] Purtle, J. (2020). Systematic review of evaluations of trauma-informed organizational interventions that include staff trainings. *Trauma Violence & Abuse*, *21*(4), 725–740. 10.1177/1524838018791304 30079827

[CR24] R Core Team (2024). _R: A Language and Environment for Statistical Computing. R Foundation for Statistical Computing, Vienna, Austria. <https://www.R-project.org/>

[CR25] Schriesheim, C. A., & Hill, K. D. (1981). Controlling acquiescence response bias by item reversals: The effect on questionnaire validity. *Educational and Psychological Measurement*, *41*(4), 1101–1114.

[CR26] Stokes, Y., Lewis, K. B., Tricco, A. C., Hambrick, E., Jacob, J. D., Demery Varin, M., & Graham, I. D. (2024). Trauma-informed care interventions used in pediatric inpatient or residential treatment mental health settings and strategies to implement them: A scoping review. *Trauma Violence & Abuse*, *25*(3), 1737–1755. 10.1177/15248380231193444 PMC11155220 37694809

[CR27] Substance Abuse and Mental Health Services Administration (2014). SAMHSA’s Concept of Trauma and Guidance for a Trauma-Informed Approach. HHS Publication No. (SMA) 14-4884. Rockville, MD: Substance Abuse and Mental Health Services Administration.

[CR28] Sundborg, S. A. (2019). Knowledge, principal support, self-efficacy, and beliefs predict commitment to trauma-informed care. *Psychological Trauma: Theory Research Practice and Policy*, *11*(2), 224. 30321020 10.1037/tra0000411

[CR29] Swain, S. D., Weathers, D., & Niedrich, R. W. (2008). Assessing three sources of misresponse to reversed likert items. *Journal of Marketing Research*, *45*(1), 116–131.

[CR30] Taber, K. S. (2018). The use of cronbach’s alpha when developing and reporting research instruments in science education. *Research in Science Education*, *48*, 1273–1296.

[CR31] Text - (2018, February 9). H.R.1892–115th Congress (2017–2018): Bipartisan Budget Act of 2018. https://www.congress.gov/bill/115th-congress/house-bill/1892/text

[CR33] Zhang, X., Noor, R., & Savalei, V. (2016). Examining the effect of reverse worded items on the factor structure of the need for cognition scale. *PloS One*, *11*(6), e0157795. 27305001 10.1371/journal.pone.0157795 PMC4909292

[CR32] Zhang, S., Conner, A., Lim, Y., & Lefmann, T. (2021). Trauma-informed care for children involved with the child welfare system: A meta-analysis. *Child Abuse & Neglect*, *122*, 105296. 34478999 10.1016/j.chiabu.2021.105296

